# Multicenter comparative study of open, laparoscopic, and robotic pyeloplasty in the pediatric population for the treatment of ureteropelvic junction obstruction (UPJO)

**DOI:** 10.1590/S1677-5538.IBJU.2022.0194

**Published:** 2022-08-20

**Authors:** Sebastian Tobía González, Tiago E. Rosito, Anna Bujons Tur, Javier Ruiz, Rafael Gozalbez, Anabella Maiolo, Patric M. Tavares, Antonio Rebello Horta Gorgen, Erika Llorens de Kencht, Yesica Quiroz Madarriaga, Santiago Weller, Ignacio Pablo Tobia, Miguel Castellan, Juan Pablo Corbetta

**Affiliations:** 1 Universidad Nacional de la Plata Department of Urology La Plata Buenos Aires Argentina Department of Urology, Universidad Nacional de la Plata – UNLP, La Plata, Buenos Aires; 2 Hospital Interzonal Especializado en Pediatría “Sor María Ludovica” Department of Urology La Plata Buenos Aires Argentina Department of Urology, Hospital Interzonal Especializado en Pediatría “Sor María Ludovica”, La Plata, Buenos Aires, Argentina; 3 Hospital de Clínicas de Porto Alegre Grupo de Urologia Reconstrutiva e Infantil Departamento de Urologia Porto Alegre RS Brasil Departamento de Urologia, Grupo de Urologia Reconstrutiva e Infantil (GURI), Hospital de Clínicas de Porto Alegre, Porto Alegre, RS, Brasil; 4 Universidade Federal do Rio Grande do Sul Programa de Pós-graduação em Ginecologia e Obstetrícia Porto Alegre RS Brasil Programa de Pós-graduação em Ginecologia e Obstetrícia, Universidade Federal do Rio Grande do Sul, Porto Alegre, RS, Brasil; 5 Fundació Puigvert Department Pediatric Urology Barcelona Catalunya Spain Department Pediatric Urology, Fundació Puigvert, Barcelona, Catalunya, Spain; 6 Ciudad Autonoma de Buenos Aires Hospital Pediatric Prof. Dr. Juan P. Garrahan Department of Urology Buenos Aires Argentina Department of Urology, Hospital Pediatric Prof. Dr. Juan P. Garrahan, Ciudad Autonoma de Buenos Aires, Buenos Aires, Argentina; 7 Nicklaus Children's Hospital Division of Pediatric Urology Miami FL United States Division of Pediatric Urology, Nicklaus Children's Hospital, Miami, FL, United States

**Keywords:** Ureteral Obstruction, Laparoscopy, Robotics

## Abstract

**Introduction:**

Dismembered open pyeloplasty described by Anderson and Hynes is the “gold standard” for the treatment of ureteropelvic junction obstruction. The aim of our study was to compare the results of open (OP) vs laparoscopic (LP) vs robotic (RALP) pyeloplasty.

**Material and Methods:**

A multicenter prospective review was conducted of pyeloplasty surgeries performed at five high-volume centers between 2014 and 2018. Demographic data, history of prenatal hydronephrosis, access type, MAG3 renogram and differential renal function, surgery time, length of hospital stay, and complication rate (Clavien-Dindo) were recorded. Access type was compared using the Kruskal-Wallis, Chi-square, or Fisher's exact tests.

**Results:**

A total of 322 patients were included: 62 OP, 86 LP, and 174 RALP. The mean age was 8.13 (r: 1-16) years, with a statistically significant lower age (mean 5 years) in OP (p < 0.001). There were no significant differences in the distribution of the side affected. Operative time was 110.5 min for OP, 140 min for LP, and 179 min for RALP (p < 0.0001). Hospital stay was significantly shorter in the RALP group than in the other groups (p < 0.0001). There were no differences in postoperative complications and reoperations between the three groups.

**Conclusions:**

Minimally invasive surgery for the management of UPJO in children is gaining more acceptance, even in patients younger than 1-year-old. Operative time continues to be significantly shorter in OP than in LP and RALP. Hospital stay was shorter in RALP compared to the other techniques. No differences were found in complication rates, type of complications, and reoperation rate.

## INTRODUCTION

Open dismembered pyeloplasty (OP), originally described by Anderson and Hynes, is the most commonly performed surgical procedure for the treatment of ureteropelvic junction obstruction (UPJO), with long-term success rate of around 95% (1, 2). Over the last two decades, however, minimally invasive surgery (MIS) techniques such as laparoscopic pyeloplasty (LP) and robot-assisted laparoscopic pyeloplasty (RALP) have been developed and popularized as a standard of care in common practice.

Since its first description by Peters et al., laparoscopic pyeloplasty has not been as popular in pediatric urology as in the adult population possibly due to its technical difficulty and long learning curve, and better recovery in children compared to adults (3, 4).

Although MIS techniques are associated with a longer operative time compared to open procedures, they have shown benefits in terms of shorter postoperative hospital stay and lower morbidity (4–6). Nevertheless, LP is technically demanding and has a long learning curve (7, 8). RALP has reduced the technical difficulty of this procedure with a shorter learning curve compared to LP (9–13). Therefore, we believe that the three techniques should have similar outcomes.

The aim of this study was to compare outcomes in safety and effectiveness between OP, LP, and RALP in a large, multicenter cohort of pediatric patients with UPJO.

## MATERIAL AND METHODS

The study was approved by the Ethics Committee of each Hospital (IRB: 18967819.8.0000.5327).

A multicenter retrospective cohort study was conducted with prospectively collected data. We included pyeloplasty surgeries (including redo cases) performed in children younger than 16 years of age between 2014 and 2018. The choice of OP, LP, and RALP were due to the surgeon and center preference, and the techniques were compared in terms of safety and effectiveness.

After IRB approval, medical records of all patients with a history of UPJO were reviewed evaluating the following characteristics: age at surgery, sex, affected side, history of urinary tract infection (UTI), flank pain, operative time (skin incision to skin closure -port removal in LP and RALP -, in minutes), type of stent placement during the surgery and length of hospital stay (determined by the computerized time recorded on hospital admission and discharge records).

Indications for surgical intervention were impairment of differential renal function (< 40%) by nuclear scan or a decrease in split renal function of > 10% on subsequent studies, obstructive drainage curve on diuretic renogram, symptomatic obstruction (recurrent flank pain, UTI), and progressive worsening of hydronephrosis on ultrasound images (urinary tract dilation - UTD - classification system) (14). Each institution's registered technique is routinely performed outside the learning curve.

Complications were assessed using the Clavien-Dindo grading system (15). Post-surgical patients were followed-up with ultrasonography every 3 months during the first year of surgery, every 6 months in the second year, and then on an annual basis. Patients were defined as having a good outcome when SFU grade hydronephrosis improved without symptom recurrence. In patients in whom SFU grade decreased but was still greater than 2, we performed a MAG3 renal scan to rule out obstruction.

Continuous variables are expressed as mean, or median and range and categorical variables are expressed as absolute value and/or percentage. For comparison between techniques, the Kruskal-Wallis, Chi-square, or Fisher's exact tests were used depending on the case. In the case of multiple comparisons, they were adjusted using the Bonferroni correction method. In all cases, a p-value of less than 0.05 was considered as a cut-off for a significant value. SPSS 22.0™ software was used.

### Surgical technique

- OP: a subcostal flank or dorsal lumbotomy approach was used. Conventional dismembered pyeloplasty was performed with a running polydioxanone (6/0 PDS® II) anastomotic suture. The bladder catheter was removed 24 hours later. A ureteral stent or nephrostomy tube was used only in select cases and a perirenal drain was left in place in all patients.
- LP: transperitoneal dismembered pyeloplasty was performed using 3 ports (one 5- or 10-mm port for the 30-degree optics and two 3- or 5-mm ports for the instruments). A running (5/0 PDS® II) anastomotic suture was placed, and a ureteral double J stent was placed in an antegrade fashion in all patients. The Foley catheter was removed on postoperative day 1 or on the next morning.
- RALP (4 trocars): an infraumbilical port was used for the optics (8.5–12 mm), an 8-mm working port in the epigastric region, another 8-mm working port below the optic port laterally to the midline in the right / left iliac fossa, and an assistant port (5 mm) was placed between the optic port and the lower working port (left pyeloplasty) or between the optics and the upper working port to lift the liver (right pyeloplasty) for the introduction of the suture, retraction, or suction.

RALP (3 trocars): 3 midline trocars (8 mm) were used, one for the camera at the umbilicus and the others at least 4 cm above and below the umbilicus, respectively.

The bladder catheter was removed 24 hours later. Ureteral stents, when placed, were removed approximately 4 weeks postoperatively via cystoscopy using short-acting anesthesia. In a group of patients in whom the stent was placed in a retrograde fashion and a string was left in the genitalia, the stent was removed in the clinic 2-3 weeks postoperatively.

## RESULTS

A total of 322 patients were included in the study: 62 OP, 86 LP, and 174 RALP. The mean age was 8.1 years (range 1-16). Children that underwent OP were younger than those undergoing procedures with the other techniques (p < 0.001 – Table-1). Demographic data analysis showed that in the RALP group female patients prevailed (p < 0.001). There were no significant differences regarding the distribution of the affected side between the three groups (Table-1).

**Table 1:** Sex, Age and Side of UPJO of patients submitted to open, laparoscopic and robotic pyeloplasty.

		OP (n=62)	LP (n=87)	RALP (n= 161)	p
**Sex: Female (%)**		18 (29)	31 (36)	95 (54,6)	0.0001
**Age: mean (range)**		5 (1 – 15)	10.5 (1-16)	8.9 (1 – 16)	0.0001
	< 1 year (%)	20 (32.3)	2 (2.3)	9 (5.2)	
	1-6 years (%)	22 (35.5)	13 (15.1)	50 (28.7)	
	> 6 years (%)	20 (32.3)	71 (82.6)	115 (66.1)	
**Side (%)**					0.469
	Right	27 (43.5)	29 (33.7)	74 (42.5)	
	Left	35 (56.5)	57 (66.3)	98 (56.3)	
	Bilateral	0	0	2 (1.1)	

**OP** = Open Pyeloplasty; **LP** = Laparoscopic Pyeloplasty; **RALP** = Robotic-assisted laparoscopic pyeloplasty

The majority of the patients were older than 6 years (64%, 206 patients). Table-1 shows the most used technique according to age group; OP was more commonly used in children younger than 1 year and LP in patients older than 6 years. In the intermediate group, OP and RALP were more commonly used than LP.

**Figure 1:**
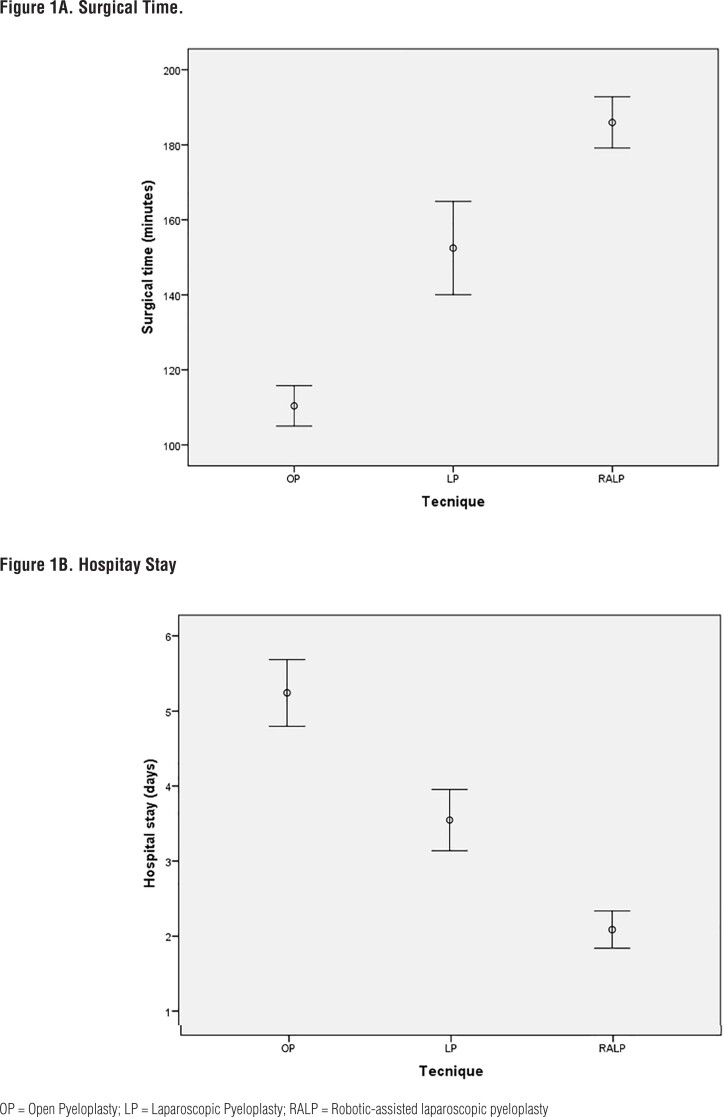
Comparison of surgical time and hospital stay in open, laparoscopic and robotic pyeloplasty.

Operative time was shorter in the OP group (110.48 min vs 140 min for LP and 179 min of RALP). On the other hand, hospital stay was shorter in the RALP group in comparison with the LP and OP groups (p < 0.0001). There were no significant differences in postoperative complications or reoperation rate. A ureteral stent was placed during surgery in almost all patients undergoing MIS, exceeding the rate of stent placement in OP (p <0.0001) (Figure-1 and Table-2).

**Table 2:** Surgery outcomes and complications’ rate of patients submitted to open, laparoscopic and robotic pyeloplasty.

	OP (n=62)	LP (n=86)	RALP (n=174)	p
**Transfusion (%)**	0	0	0	NS^1^
**Operative time, median (min.)**	110 (60-210)	140 (60-316)	179 (108-361)	p = 0.0001
**Stent (%)**	22 (35.5%)	83 (96.50%)	174 (100%)	p = 0.0001
**Length of hospital stay, median (days)**	5 (3 – 13)	3 (1-12)	1 (1-11)	p = 0.0001
**UPJO recurrence (n)**	1	2	2	NS
**Follow-up months median (range)**	57.8 (7-136)	41.1 (3-87)	44.6 (3-140)	

OP = Open Pyeloplasty; LP = Laparoscopic Pyeloplasty; RALP = Robotic-assisted laparoscopic pyeloplasty; UPJO = Ureteropelvic Junction Obstruction

NS = non-significant

In only 5 patients (1.55%) UPJO recurred after a median follow-up of 42.3 months. In these patients, SFU grade decreased but was still greater than 2. MAG3 renal scan confirmed the obstruction (Table-2).

When analyzing complication rate (n=51) according to initial vs redo procedures, Clavien-Dindo grades IIIb to V were found to be mostly associated with redo surgeries with a statistically significant difference.

## DISCUSSION

In our study, RALP and LP proved to be safe and successful methods, with several advantages over OP. While operative time was longer, hospital stay was shorter. As shown in previous studies (16, 17) operative time was longer in RALP than in LP and OP. A contributing factor may have been older age, which has been shown to be associated with operative time (16) in the RALP group.

The first important outcome to be considered after minimally invasive pyeloplasty is the success of the procedure. In a meta-analysis, Cundy et al. compared RALP, LP, and OP in children. Twelve articles were included of which five were cohort and seven case-controlled studies. Success rates of higher than 95% were reported for RALP and LP, whereas in the comparative studies of RALP and OP included, success rates were higher than 87% and the procedures were found to be comparable (18). Another meta-analysis by Huang et al. compared the outcomes of LP and OP in children. One randomized controlled trial and 15 comparative studies were included in the analysis. Success rates of both procedures were between 83 and 100% and were found to be comparable (19). In our series, the success rates were higher than 95% and no statistically significant difference was observed regarding success between the procedures.

In infants, the operation may be more challenging due to the limited space; (19) however, RALP has recently been successfully performed in infants, with an operative time that was even shorter than that of OP (20, 21). Other techniques such as the Flexdex® articulating needle driver may facilitate the procedure (22).

Postoperative hospital stays of patients undergoing RALP and LP were shorter compared to open surgery. Since the children had no underlying conditions (comorbidities), the length of hospital stay was a reasonably good measure of the trauma caused by the surgery. A reduced length of hospital stay with RALP/LP has previously been shown and is of major importance, both for the patient and caregivers and for the hospital (23).

No statistical difference was found between the three groups regarding complication rate. Compared to other studies reporting complication rates of 0–33% for RALP (24), our results were in the lower range (0-18%). In addition, most complications were stent-related and transient, with only 14/322 (4.3%) patients requiring a second surgical intervention. It seemed that postoperative complications, especially those related to pyelonephritis and stent morbidity, increase the risk of needing secondary repair. On the other hand, it is possible that not the complications per se increased the risk for reoperation, but that the complications were an indication of an already distorted healing process related to either the surgery or factors inherent to the patient. Complications rates were higher in the redo surgeries group, but some studies showed that other techniques such as endopielolitotomy have a greater failure rate (25).

In a prospective comparative case-control study (OP vs LP), Piaggio et al. (26) described four complications in the LP group: febrile UTI (two), double J stent disruption, and meatal stenosis (one each); and three in the OP group: febrile UTI, flank pain due to stent displacement, and persistent gross hematuria (one each). Two patients in the LP group and one in the OP group needed additional procedures (p > 0.05): meatoplasty and ureteroscopy for stent removal (LP) and stent repositioning (OP).

The optimal age is one of the concerns for the use of LP in children. It is generally believed that it is difficult to perform LP in small children due to the risk of pelvic perforation when introducing the trocars. Excellent intracorporeal laparoscopic suturing skills are imperative in limited spaces (27). In our series, no significant differences were found when comparing reoperations and/or complications according to age.

In a systematic review, only two studies presented the age of the patient as mean +- SD (28). The analysis showed that patients who underwent LP were significantly older (50.90 months) than those who underwent OP. The authors of the seven remaining studies also found that patients in the LP group were older than those in the OP group. Other authors, however, reported similar or even younger ages in the LP group compared to the OP group (29, 30).

The main strength of this international multicenter study is the large number of patients showing good effectiveness of the procedure regardless of the technique (OP, LP, or RALP) or age. No significant differences were found when comparing with reoperations and/or complications according to age.

This study has some limitations, especially due to the retrospective design, but this is a multicenter study in the Ibero-american scenario with a significant number of patients. The difference in age between the groups could be a selection bias that could affect the results. Also, in pediatric urology controversy remains regarding what patients with hydronephrosis require surgery and what clinical and imaging methodology should be selected to define recurrence of UPJO.

## CONCLUSIONS

Open, laparoscopic, and robotic-assisted pyeloplasty in the pediatric population have similar success rates and are safe to perform, even in children less than 1-year-old where traditionally open pyeloplasty is more performed. No differences were found in complication rates, type of complications, and reoperations between the three groups. Operative time was shorter in the OP. Severe complications required a greater number of reoperations.

More studies are needed to evaluate RALP, especially in infants. In addition, aspects, such as surgical costs and patient satisfaction, should be further assessed.
